# Transoral Robotic Surgical Resection of Bilateral Parapharyngeal Space Rhabdomyoma

**DOI:** 10.1155/crot/2786874

**Published:** 2025-12-12

**Authors:** Jacob S. Brady, Craig Miller, Sonya Ahuja, David M. Rogers, Zain H. Rizvi

**Affiliations:** ^1^ Department of Otolaryngology-Head and Neck Surgery, Case Western Reserve University, Cleveland, Ohio, USA, case.edu; ^2^ Department of Otolaryngology-Head & Neck Surgery, Virginia Mason Medical Center, Seattle, Washington, USA, virginiamason.org; ^3^ Pathology and Laboratory Medicine Service, Veterans Affairs Puget Sound Health Care System, Seattle, Washington, USA, va.gov; ^4^ Department of Otolaryngology-Head & Neck Surgery, University of Washington, Seattle, Washington, USA, washington.edu; ^5^ Department of Surgery, Veteran Affairs Puget Sound Health Care System, Seattle, Washington, USA

**Keywords:** head and neck surgery, parapharyngeal space, robotic surgery, skull-based surgery, tumors of the head and neck

## Abstract

**Background:**

The parapharyngeal space is a surgically challenging anatomic compartment with critical neurovascular structures with limited access options that often requires an open transcervical skull base approach, often in combination with parotidectomy and/or a transoral approach. As such, lesions in this area must be carefully approached and represent a challenge even for experienced surgeons. Isolated transoral approaches have been used but are often limited by visualization of the deeper structures of the parapharyngeal space. While transoral robotic resection has been used to approach the parapharyngeal space, it must be performed after careful patient selection.

**Case Presentation:**

We present a patient with massive bilateral rhabdomyoma of the parapharyngeal space who presented following dysphagia. A minimally invasive transoral approach was used to successfully remove both tumors simultaneously. This patient successfully underwent removal of bilateral tumors using a completely transoral robotic‐assisted approach. The patient experienced no long‐term sequela including dysphagia, bleeding, cranial neuropathy, and airway distress. A literature search regarding bilateral parapharyngeal space tumors and transoral approaches to the parapharyngeal space was performed to examine the rarity, safety, and efficacy of the approach. This represents the only report of such pathology as well as the only report of a simultaneous bilateral parapharyngeal space approach with transoral robotic surgical assistance.

**Conclusion:**

Bilateral parapharyngeal space tumors are an incredibly rare phenomenon. Following careful patient selection and postoperative monitoring, removal of bilateral tumors can be performed in a safe and successful manner without complication, morbidity, or external incision.

## 1. Introduction

Rhabdomyomas are a rare and benign neoplasm consisting of either differentiated skeletal or cardiac muscle [1]. Within the head and neck, they are quite rare overall, accounting for approximately 0.5% of all head and neck masses. However, among adults with extracardiac rhabdomyoma, 90% of these masses are found in the head and neck [2]. Even more uncommon are multifocal head and neck rhabdomyoma [3]. A 2020 systematic review found only 29 cases was reported in the literature worldwide [4]. Of these, the most common locations within the head and neck were the submandibular and parapharyngeal spaces (PPSs). The PPS is a complex anatomical area with distinct surgical concerns, as it contains the internal carotid artery, internal jugular vein, cranial Nerves IX–XII, and the cervical sympathetic chain, all of which are vulnerable to injury during tumor resection [5]. With a very low potential for malignant transformation, the primary concern for head and neck rhabdomyomas is a mass effect on adjacent structures with resultant dysphagia, dysphonia, or airway compromise. Traditionally, removal of these masses, particularly those in the PPS, is reserved for symptomatic patients and performed via a *trans*‐cervical, *trans*‐parotid, or *trans*‐mandibular approach [5]. With the advent of transoral robotic surgery (TORS), the lesions accessible via a *trans*‐oral approach is ever expanding and now includes some PPS lesions [6–12]. Presented here is a case of a bilateral parapharyngeal rhabdomyoma which was completely removed via a TORS approach and without any significant short‐ or long‐term complications and a relatively short hospital stay.

## 2. Case Presentation

A 77‐year‐old male with a 19‐year history of a known right pharyngeal mass re‐presented following increasing dysphagia. His PPS lesion was identified in 2001 as a rhabdomyoma on operative biopsy, after two separate fine‐needle aspirations (FNAs) were nondiagnostic. At that time, the patient elected to monitor the mass as his symptoms were minimal, and the pathology was benign. However, over the following 19 years, the mass continued to grow resulting in worsening dysphagia, intermittent dyspnea on exertion without respiratory distress, and worsening snoring. He therefore re‐presented for further management. He otherwise denied new neck masses, weight loss, stridor, and constitutional symptoms. He was otherwise healthy with no significant comorbidities or allergies apart from a 40‐pack‐year smoking history. On examination, the patient had a large, soft, submucosal mass along the right oropharynx with a deviated uvula to the left and a patent left oropharynx. All cranial nerves were intact; he had no cervical lymphadenopathy, and the remainder of his head and neck examination was without abnormality. Flexible nasopharyngolaryngoscopy showed a mass emanating from the right tonsillar/parapharyngeal region that extended down to the hypopharynx. The mass extended to the supraglottic region, but the larynx was clear of any involvement or mass effect, and the bilateral vocal cords were mobile. A CT and MRI neck with contrast (Figure 1) were performed which revealed a large, multiloculated, prestyloid right parapharyngeal mass extending from the skull base down to the level of the suprahyoid epiglottis and laterally to the deep parotid lobe with preserved fat planes. These scans also revealed a smaller, left, PPS mass, similar in appearance to the right‐sided mass. Given his prior history of nondiagnostic FNA and need to obtain a higher yield diagnostic sample, an in‐office incisional biopsy was performed following consultation with pathology to confirm the diagnosis and ensure that no malignant transformation had occurred in the interval 19‐year period. This pathology was consistent with rhabdomyoma. Given the worsening dysphagia and dyspnea and the desire for definitive management, the patient elected to proceed with surgical resection. A discussion of transoral robotic approach with possible transcervical approach was discussed, and he was deemed a candidate for a primary transoral robotic approach after his clinical exam showed > 3 cm mouth opening, no mandibular or maxillary tori, full range of neck motion and neck extension, and imaging that did not demonstrate a retropharyngeal carotid artery.

Figure 1(a) Axial CT. (b) Coronal CT demonstrating massive bilateral parapharyngeal space tumors.

**Figure:**
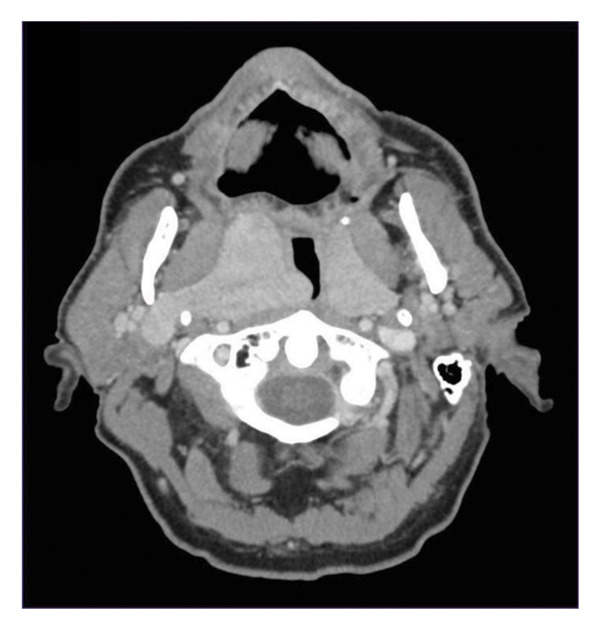
(a)

**Figure:**
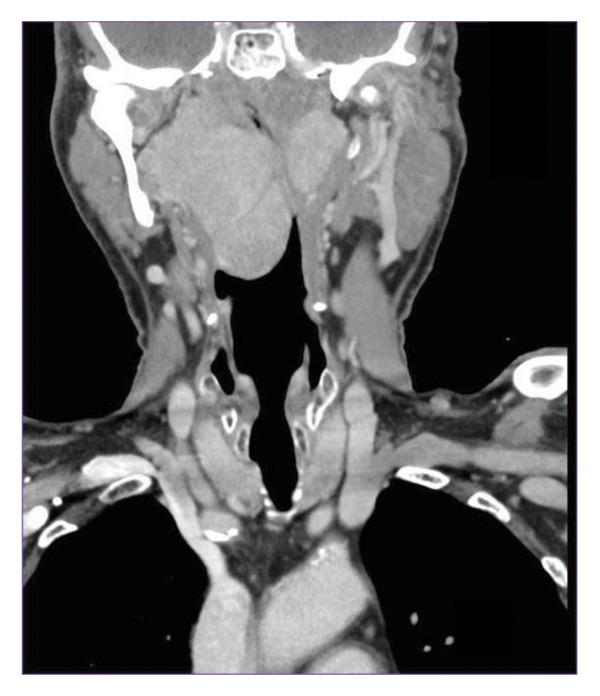
(b)

## 3. Surgical Management, Hospital Course, and Outcome

After informed consent was confirmed, the patient was brought to the operating room and was placed under general anesthesia with nasotracheal intubation. A moldable mouth guard was used on the upper alveolus, and an FK retractor was inserted into the oral cavity to retract the tongue and jaw. The Da Vinci Xi system was then moved into place and targeted into the oral cavity. A 0° endoscope was used initially as well as a Maryland dissector and bovie electrocautery.

The right side was first addressed (Figure 2(a)). A vertical incision was made between the palatoglossus and palatopharyngeus muscles. The dissection was deepened through the constrictor muscles until the mass was identified. The mass was circumferentially dissected and retracted medially. Superiorly, the lateral aspect of the mass was traced around the stylomandibular ligament and toward the deep parotid gland. The glossopharyngeal nerve was identified, preserved, and gently retracted during this approach. The mass was carefully teased out, predominantly using blunt dissection. The internal maxillary artery and its terminal branches were identified, clipped, and ligated (Figure 2(b)). Due to the nasopharyngeal component of the tumor, the initial mucosal incision was carried further superiorly into the soft palate, approximately 1 cm. This allowed improved visualization of the nasopharynx. A 30° endoscope was then placed into the Da Vinci robot and turned toward the nasopharynx. Dissection was then carried circumferentially into the superior most extent of the PPS at the level of the skull base. Once the mass was completely removed of any attachments, it was delivered into the oropharynx and removed transorally (Figure 2(c)). The pharyngotomy repaired and palatal incision reapproximated using 3‐0 Vicryl in an interrupted horizontal mattress fashion.

Figure 2(a) Fullness of the parapharyngeal space viewed transorally. (b) Clipping the internal maxillary artery. (c) Delivery of tumor to the oropharynx.

**Figure:**
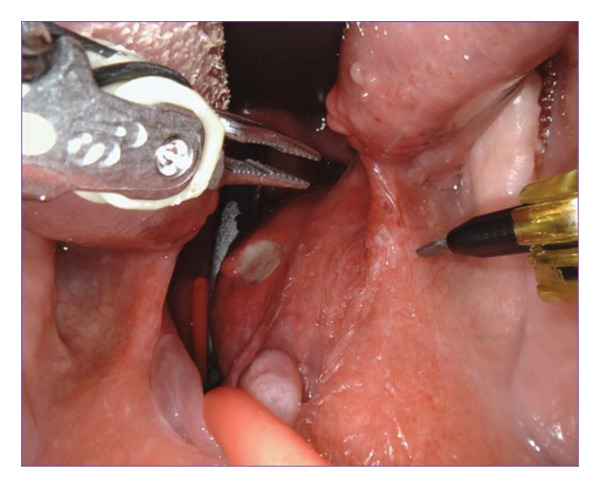
(a)

**Figure:**
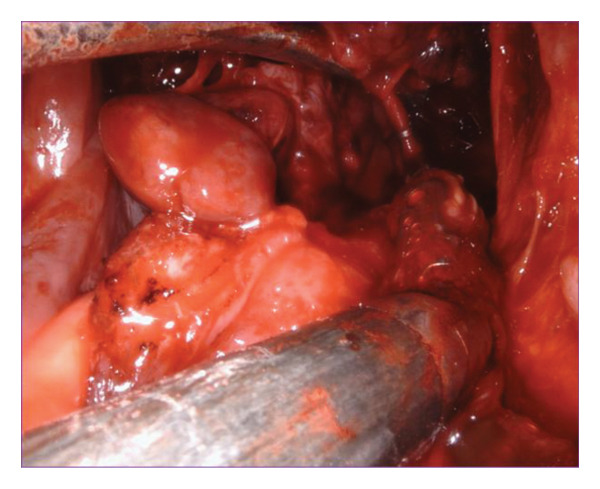
(b)

**Figure:**
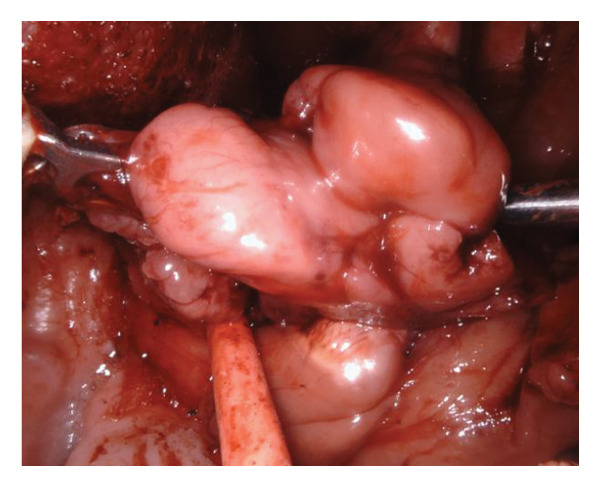
(c)

Attention was then turned to the left side where a vertical incision was made again in the same area as on the right and deepened through the constrictor muscles. The mass was identified and retracted. This smaller mass required minimal blunt dissection to deliver the tumor into the oropharynx. Once this was completely removed, the pharyngotomy was repaired in a similar fashion. After careful examination of his pharynx, the anticipated postoperative edema was felt to not necessitate a tracheostomy. A nasogastric tube was also deemed unnecessary as the postoperative dysphagia was anticipated to be short, and the patient was to be started on an oral diet immediately without concern for the surgical bed as is routine for TORS at our institution. The patient was extubated successfully in the operating room.

Postoperatively, the patient was electively admitted to the surgical intensive care unit for close airway monitoring and was transferred to the ward on postoperative Day (POD) 1. His pain was well managed with scheduled ibuprofen and acetaminophen, as well as oxycodone as needed for analgesia. His dysphagia was initially profound but improved dramatically with a 24‐h course of intravenous dexamethasone, and he was quickly tolerating an oral diet. His postoperative course was otherwise unremarkable, and he was discharged on POD 4 on a soft diet. The patient was seen postoperatively at 1 and 4 weeks. At his last follow‐up, his pain was completely resolved, was tolerating a normal diet, had no wound complications, and all initial presenting symptoms completely resolved. At 6‐month follow‐up, he remained asymptomatic but declined further radiographic or clinical follow‐up. Final pathology ultimately showed the right‐sided mass to be a homogenous, pink‐tan, smooth, glistening, slightly hemorrhagic lesion measuring 7.8 × 8.1 × 4.5 cm and weighing 71.1 g. The left was similar in appearance, measuring 3.6 × 4.2 × 3.3 cm, and weighing 14.9 g. Microscopic examination showed rhabdomyoma bilaterally (Figure 3).

Figure 3(a) Histology of rhabdomyoma showing sheets of round skeletal muscle cells with eosinophilic, vacuolated cytoplasm and small nuclei (hematoxylin and eosin, 10x). (b) Higher‐magnification view showing focally present but not well‐developed sarcomeric bands on hematoxylin and eosin stain (hematoxylin and eosin, 40x).

**Figure:**
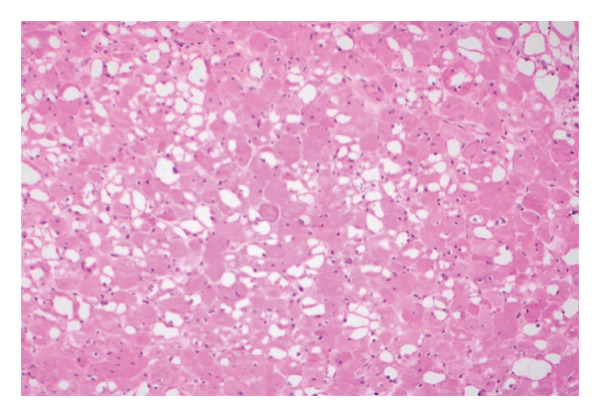
(a)

**Figure:**
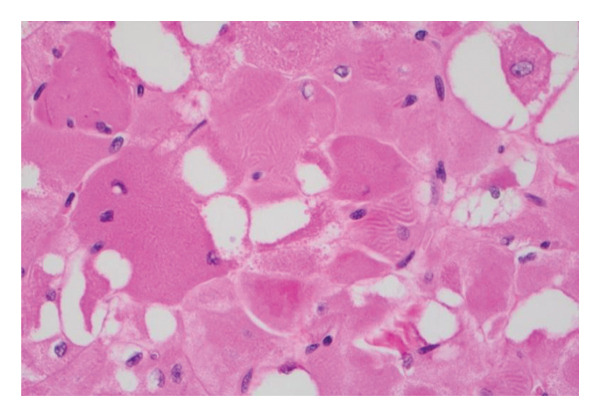
(b)

## 4. Discussion

Parapharyngeal rhabdomyoma is a rare clinical entity in a particularly complex region of the head and neck and skull base. Bilateral PPS rhabdomyoma are even more uncommon, with only 2 patients identified on systematic review [4]. The risks of surgery in this area are not insignificant and include large‐volume hemorrhage, persistent cranial neuropathies with resultant dysphonia or dysphagia, and airway distress. In the case of bilateral tumors, these risks are increased with the potential for significant impact on the aerodigestive tract. Traditional approaches have required large external incisions, parotid and facial nerve dissection, or mandibulotomy. The transoral approach has been used to access the PPS but has traditionally thought to be limited in both visualization and dissection laterally. However, a 2020 series of 5 cases showed complete excision of 2 poststyloid and 3 prestyloid benign PPS lesions with no recurrence and a single case of Horner’s syndrome [10]. In the case reported here, TORS techniques were used to remove bilateral PPS rhabdomyoma under the same anesthetic course, the largest of which extended up into the nasopharynx, and down into the hypopharynx, measuring over 8 cm. Complete resection was achieved without any short‐ or long‐term complications, airway distress, external incisions, and with complete preservation of swallowing.

Previous reports demonstrated adequate and safe resection of benign PPS masses using TORS. A recent systematic review by De Vergilio et al. found that out of 113 cases of PPS mass resections which utilized TORS, only 18 required the use of an external approach for complete resection, none of the patients has persistent cranial neuropathies, and 95.5% of patients had no persistent dysphagia [8]. Another consideration may be tumor capsule preservation, particularly in the context of other benign lesions of the PPS such as pleomorphic adenoma, where capsule violation may increase the risk of recurrence. In previous studies, the rate of capsule violation with a TORS approach to the PPS has ranged from 14% to 24% and is at least comparable to traditional external approaches [8, 9, 11, 12]. However, this case represents a particularly unique challenge due to the bilateral tumors, both of which were amenable to simultaneous TORS resection. We elected to proceed with resection of the smaller left‐sided lesion following successful extirpation of the right tumor, as the impact on swallowing and airway caliber from further PPS dissection and edema would be offset by the improvement in the physical dimension of the aerodigestive tract following surgery.

To our knowledge, this is the first ever reported case of bilateral PPS masses, of any histology, that were amenable to simultaneous transoral resection. This was accomplished without any significant complication, morbidity, or external incisions. It should be noted that this case, like the majority of previous studies and reports, focuses on a benign disease process without carotid encasement, bony erosion, or otherwise malignant features which would alter the surgical approach and may require a combination with an external approach. This suggests that the removal of bilateral massive tumors is safe using a transoral approach for this rare benign entity.

## Consent

This study was reviewed, and the need for informed consent was waived by the Veterans Affairs Puget Sound Office of Research and Development. All patient identifiers were removed and the case anonymized as per ICMJE guidelines.

## Conflicts of Interest

The authors declare no conflicts of interest.

## Author Contributions

Study design, manuscript preparation, and review: Zain H. Rizvi and Jacob S. Brady; manuscript preparation and review: David M. Rogers, Craig Miller, and Sonya Ahuja.

## Funding

The authors received no specific funding for this work.

## Data Availability

The data are readily available upon request from the corresponding author.

## References

[1] Dermawan J. K. , Doxtader E. , Chute D. J. , and Policarpio-Nicolas M. L. , Cytologic Findings of an Adult Rhabdomyoma in the Parapharyngeal Space: A Report of a Case and Review of the Literature, Diagnostic Cytopathology. (2018) 46, no. 5, 419–424, 10.1002/dc.23860, 2-s2.0-85033554904. 29131558

[2] Papaspyrou G. , Werner J. A. , Roessler M. , Devaney K. O. , Rinaldo A. , and Ferlito A. , Adult Rhabdomyoma in the Parapharyngeal Space: Report of 2 Cases and Review of the Literature, American Journal of Otolaryngology. (2011) 32, no. 3, 240–246, 10.1016/j.amjoto.2010.01.007, 2-s2.0-79955523654. 20392534

[3] Allevi F. , Rabbiosi D. , Colletti G. et al., Extensive Rhabdomyoma of the Head and Neck Region: A Case Report and a Literature Review, Minerva Stomatologica. (2013) 62, no. 10, 387–395. 24217686

[4] Khalaf M. G. , Haddad R. , Akiki M. , Khazen J. , and Melkane A. E. , Multifocal Adult Rhabdomyoma of the Head and Neck: Case Report and Systematic Review of the Literature, International Journal of Oral and Maxillofacial Surgery. (2021) 50, no. 3, 327–334, 10.1016/j.ijom.2020.07.018. 32773112

[5] Olsen K. D. , Tumors and Surgery of the Parapharyngeal Space, The Laryngoscope. (1994) 104, no. 5, 1–28, 10.1288/00005537-199405000-00001, 2-s2.0-0028236882. 8189998

[6] Meulemans J. , Delaere P. , and Vander Poorten V. , Early Experience in Transoral Robotic Surgery (TORS) for Non-Oropharyngeal Head and Neck Malignancies: A Review of Functional and Oncologic Outcomes, B-ENT. (2015) 24, 21–31. 26891528

[7] Park Y. M. , De Virgilio A. , Kim W. S. , Chung H. P. , and Kim S. H. , Parapharyngeal Space Surgery via a Transoral Approach Using a Robotic Surgical System: Transoral Robotic Surgery, Journal of Laparoendoscopic & Advanced Surgical Techniques. (2013) 23, no. 3, 231–236, 10.1089/lap.2012.0197, 2-s2.0-84879088500. 23343202

[8] De Virgilio A. , Costantino A. , Mercante G. , Di Maio P. , Iocca O. , and Spriano G. , Trans-Oral Robotic Surgery in the Management of Parapharyngeal Space Tumors: A Systematic Review, Oral Oncology. (2020) 103, 10.1016/j.oraloncology.2020.104581. 32058293

[9] O’Malley B. W. , Quon H. , Leonhardt F. D. , Chalian A. A. , and Weinstein G. S. , Transoral Robotic Surgery for Parapharyngeal Space Tumors, ORL; Journal for Oto-Rhino-Laryngology and Its Related Specialtie. (2010) 72, no. 6, 332–336, 10.1159/000320596, 2-s2.0-77957298131. 20924206

[10] Panda S. , Sikka K. , Thakar A. , Sharma S. C. , and Krishnamurthy P. , Transoral Robotic Surgery for the Parapharyngeal Space: Expanding the Transoral Corridor, J Robot Surg. (2020) 14, no. 1, 61–67, 10.1007/s11701-019-00932-3, 2-s2.0-85061599357. 30762172

[11] Maglione M. G. , Guida A. , Pavone E. et al., Transoral Robotic Surgery of Parapharyngeal Space Tumours: A Series of Four Cases, International Journal of Oral and Maxillofacial Surgery. (2018) 47, no. 8, 971–975, 10.1016/j.ijom.2018.01.008, 2-s2.0-85041354916. 29397299

[12] Chan J. Y. , Tsang R. K. , Eisele D. W. , and Richmon J. D. , Transoral Robotic Surgery of the Parapharyngeal Space: A Case Series and Systematic Review, Head & Neck. (2015) 37, no. 2, 293–298, 10.1002/hed.23557, 2-s2.0-84921435421. 24288351

